# Correlation between luteinizing hormone receptor gene expression in human granulosa cells with oocyte quality in poor responder patients undergoing *in vitro* fertilization: A cross-sectional study

**DOI:** 10.12688/f1000research.17036.1

**Published:** 2019-01-04

**Authors:** Budi Wiweko, M. Luky Satria, Kresna Mutia, Pritta Ameilia Iffanolida, Achmad Kemal Harzif, Gita Pratama, R. Muharam, Andon Hestiantoro

**Affiliations:** 1 Division of Reproductive Endocrinology and Infertility Department of Obstetrics and Gynecology, Faculty of Medicine Universitas Indonesia, Jakarta, 10430, Indonesia; 2 Yasmin IVF Clinic, Dr. Cipto Mangunkusumo General Hospital, Jakarta, 10430, Indonesia; 3 Human Reproductive, Infertility and Family Planning Research Center, Indonesia Medical Education and Research Institute(IMERI), Faculty of Medicine, Universitas Indonesia, Jakarta, 10430, Indonesia

**Keywords:** Granulosa Cells, LH-Receptor, Oocytes, Poor Responder, qRT-PCR

## Abstract

**Background: **This study was performed to evaluate the role of luteinizing hormone (LH) and granulosa cell LH receptor (LH-R) in poor responder patients who underwent controlled ovarian stimulation. Expression levels of LH-R mRNA in granulosa cells was investigated and compared with oocyte morphology, oocyte maturity and fertilization rate.

**Methods: **Granulosa cells were obtained from 30 patients who underwent *in vitro* fertilization (IVF) at Dr. Cipto Mangunkusumo Hospital, Jakarta. The patients were divided into two groups: group I (n=10) poor responders; and group II (n=20) non-poor responders. After the extraction of total RNA from granulosa cells, semi-quantitative RT-PCR was performed and the amount of LH-R mRNA was quantified. The relative values were calculated as the ratio of LH-R mRNA and actin beta mRNA. Statistical analysis was performed using Mann-Whitney test and Spearman correlation.

**Results: **The relative value of LH-R mRNA was higher in group I compared with group II (27.37[0.00-28939.37] vs 0.00[0.00-7196.12]). Oocyte maturity (r=0.267) and morphology (r=0.267) in group I consistently showed a positive correlation with LH-R mRNA; in group II a negative correlation with LH-R mRNA was shown for oocyte maturity (r= -0.552) and morphology (r= -0.164). Group I had a positive correlation between LH-R expression with fertilization rate (r=0.430), and group II showed a negative correlation (r=-0.340).

**Conclusions:** The expression of LH-R mRNA has a positive correlation with oocyte quality in poor responder patients and a negative correlation in non-poor responders. Our study suggests an optimal expression of LH- R mRNA in granulosa cells during controlled ovarian stimulation to obtain good quality oocytes.

## Introduction

In 2012, the World Health Organization reported that 80 million reproductive-aged couples, which constitutes 10% of the total number of couples globally, have issues related to infertility ^1^. In Indonesia, 12–15% of reproductive-aged couples have infertility issues ^2, 3^. One way to manage infertility issues is using assisted reproductive technology (ART). One method of ART that is commonly used is *in vitro* fertilization (IVF). The *In Vitro* Fertilization World Report 2000 showed that the average number of pregnancy and births post-IVF is 26.7% and 18.6%, respectively ^4^. According to a 2008 report, the percentage of pregnancy post-IVF in Indonesia is 20–52.9% ^5^. As reported by the Society of Assisted Reproductive Technology, the success rate of IVF in women under 35 years of age is 41–43% ^6^. However, this success rate declines as a woman’s age increases, especially for women who are older than 35 years old and those who are not adequately affected by gonadotropin treatment (also known as a poor responders) ^7^.

According to the The European Society of Human Reproduction and Embryology (ESHRE) 2011 consensus in Bologna, a poor responder is defined by the presence of 2 of 3 of the following criteria: (1) more than 40 years of age; (2) ovary reserve test such as the basal antral follicle count (BAF) <6-8 follicles/ovary, or level of anti-Müllerian hormone (AMH) <0.5-1.1 ng/mL; and (3) history of ovary stimulation producing < 3 oocytes ^8^. In the USA, approximately 80.3% cancellations of an IVF cycle are caused by an inadequate number of eggs following ovarian stimulation ^9^. Poor responders have a lower pregnancy rate compared with normal responders. Poor responders have a pregnancy rate varying from 7.6 to 17.5% compared with normal responders, varying from 25.9 to 36.7% ^10^. The failure rate among the poor responder group is quite high, caused by the very low amount of oocytes and the low quality of oocytes, which eventually affects embryo quality. Low embryo quality will cause low implantation rate and high miscarriage rate ^11, 12^. If the poor responders eventually becomes pregnant, the risk of having pregnancy complications, such as hypertension and pre-eclampsia, increases ^13^.

Luteininzing hormone (LH) is an important glycoprotein hormone that regulates gonadal function that is subsequently involved in menstrual cycle physiology. LH works through LH receptor (LH-R), which are expressed in theca, granulosa, and cumulus cells. LH will bind to LH-R that is present in the cell membrane. Although the role of LH in the non-poor responder cycle is undisputed, the role of LH in ovarian stimulation during IVF is still debatable. LH supplementation for patients that respond positively toward gonadotropin releasing hormone (GnRH) agonists does not increase the number of pregnancies ^14^. Several studies show an advantage of LH supplementation on poor responders who were using GnRH agonist ^15, 16^. In a study with Asian women who were using GnRH agonist, LH supplementation was recommended for poor responders during previous IVF cycles for slow follicle growth during days 6–8 of stimulation. This study also suggested LH supplementation for women at risk of suboptimal response, primarily those who are >35 years of age ^17^. However, like several other studies, LH supplementation in this particular group of women did not significantly affect pregnancy outcomes ^17– 19^. For poor responders who were using the GnRH antagonist protocol, LH supplementation showed a better IVF outcome ^20– 22^. However, Konig *et al.* claimed no significant difference between LH supplementation for women > 35 years of age who were using the GnRH agonist protocol ^7^. The difference between the effect due to administration of follicle stimulating hormone (FSH) and LH may be due to the difference in hormone receptor expression on oocyte cells that play a role in the maturation of the follicle ^22^.

A study by Humaidan *et al.* in 2002 reported that women who had LH levels of <0.5mlU/ml and >1.51mlU/ml on day 8 of stimulation had a lower implantation rate compared with women with LH levels between 0.5mlU/ml and 1.51 mlU/ml ^23^. This particular study showed that LH has an optimal lower and upper threshold to reach adequate growth and maturation of egg cells. However, another study by Humaidan *et al.* in 2004 showed that women with LH level > 1.99 mlU/ml also had good results after LH supplementation ^24^. This implies that there was an inadequate LH bioactivity; therefore, even though LH endogen level was within optimum range, it may not give an optimum effect. Alviggi *et al.* further suggested that an LH polymorphism (v-bethaLH) resulted in a group of women showing inadequate response after FSH administration, despite having enough ovarian reserve ^25^.

Many theories have tried to explain the etiology of poor ovarian response towards gonadotropin administration. Age and low ovarian reserve are the most common factors used to explain the presence of a poor responder patient group. However, some poor responders are still young, thus the etiology of poor responders seems to be multifactorial, including decreased blood flow to the ovary, decreased aromatase activity, FSH and LH receptor polymorphisms, and autoimmunity towards the ovary ^26^. Understanding LH-R gene expression in humans is important to increase the success rate of IVF. The importance of LH during follicular phase and the optimum dosage of LH for IVF patients are still debatable ^27^. Genetic studies have had important roles in understanding the pathogenesis of diseases and development of therapy. By understanding genetic studies with a focus on gene and gene product, IVF specialists can decide the appropriate therapy for patients ^18^. Therefore, this study investigates the correlation between LH-R granulosa expression in poor responder patients and non-poor responder patients who are going through an IVF program and compares the oocyte quality outcome, fertilization rate, and pregnancy rate.

## Methods

### Study setting

This cross-sectional study was conducted to find the correlation between LH-receptor gene expression in granulosa cells with oocyte quality in poor responder patients undergoing IVF. The study took place at Yasmin Clinic, Dr. Cipto Mangunkusumo Hospital, Jakarta, Indonesia, between January and June 2015. This study was approved by the Ethics Committee of the Faculty of Medicine, Universitas Indonesia (now called the Health Research Ethics Committee, Universitas Indonesia and Dr. Cipto Mangunkusumo Hospital (HREC-FMUI/CMH) (approval number, 631/UN2.F1/ETIK/2014).

### Participants

Women attending the Yasmin Clinic for IVF procedures were selected according to Bologna criteria based on anamnesis, ultrasound, and laboratory examination ^8^. The patients underwent ovarian stimulation, which is part of the IVF procedure, continuing to the ovum pick-up (OPU) procedure. Prior to OPU, patients were offered to participate in the study. An explanation of the research, objectives, procedures, benefits, risks and expected study outcomes were provided, along with an informed consent form. The subjects who were willing to participate in the study were asked to sign the consent form. Patients with incomplete baseline data and who failed the OPU procedure were not included in this study. In total, 30 patients were recruited from January to June 2015 in the Yasmin IVF Clinic Dr. Cipto Mangunkusumo General Hospital, Jakarta. The patients were divided into two groups: I poor responders (n=10); and II non-poor responders (n=20). The poor responder group (group I) matched minimum 2 of these following criteria: (1) more than 40 years of age; (2) ovary reserve test such as the basal antral follicle count (BAF) <6-8 follicles/ovary, or level of anti-Müllerian hormone (AMH) <0.5-1.1 ng/mL; and (3) history of ovary stimulation producing < 3 oocytes. Patients who do not have those criteria, entered into group II.

### Data collection

At the time of OPU, the doctor extracted intrafolicular fluid from the patient under anesthesia. The intrafolicular fluid containing oocytes and granulosa cells was processed by the embryologist, and then the oocyte is processed in the next stage of IVF. The granulosa cells were stored at -20°C before use.

The RNA of the granulosa cells was extracted using High Pure RNA Isolation Kit (Roche, Mannheim, Germany). Measurement of total RNA concentration was made using NanoVue spectrophotometer (General Electric). Subsequently, cDNA synthesis was performed. The positive control used was mRNA from the Transcriptor First Strand cDNA synthesis kit (Roche). A light cycler fast start DNA MasterPLUS SYBR green I (Roche) was used for real time polymerase chain reactions (PCR), with the following profile: pre-incubatiton (1 cycle at 95°C for 10 minutes), quantification (45 cycles each at 95°C for 10 seconds, 65°C for 10 seconds and 72°C for 25 seconds), melting curve (1 cycle each at 95°C for 0 seconds, 65°C for 60 seconds and 95°C for 0 seconds). All the procedures are according to the manufacturer’s instruction.

In this study, we used one microgram of complementary DNA (cDNA) per reaction in a 10 microliter reaction volume. Beta-actin RNA was chosen as a suitable nonpoorization control gene. LH-R gene quantification was done using Light Cycler Fast Start DNA MasterPLUS SYBR green I kit (Roche). The real time PCR was performed using Light- Cycler 2.0 Instrument (03531414001, Roche). Primer sequences can be seen in Table 1. Output data of qRT-PCR were used to calculate the ratio of gene LH-R is the value of delta Rn. The value is the result of fluorescence detection by qRT-PCR machine and translated by using LightCycler Software Version 4.1 (Roche). The Rn value was stored in file comma delimited file (CSV) using the Kingsoft Spreadsheet program Version 2013. The data were then processed using the R Studio program Version 2.11.1, which had been added to the qpcR software package. The result of script application is the ratio value, Cp, and the efficiency of each reaction.

**Table 1.** Real time polymerase chain reaction primer sequences.

Gene	Primer sequence (5’-3’)	Size (bp)	Accession No.
LH-R	Forward: CATTCAATGGGACGACACTG Reverse: GCCTCCAGGAGATTGACAAA	235	NM000233
B-actin	Forward: ACTCTTCCAGCCTTCCTTCC Reverse: AGCACTGTGTTGGCGTACAG	117	NM001101.3

For the oocyte morphology data, we used modification scoring system based on the Xia criteria, including first polar body, perivitelline space and cytoplasmic granulation ( Table 2) ^28^. Morphology scores were measured to all oocyte retrieved with minimum score of 0 and maximum scores of 6. Mean value of morphology scores per patient were used for analysis. The percentage of fertility rate were measured from the total of fertilized divided by total embryos.

**Table 2.** Modification of Xia morphological criteria.

Criteria		Score	
	0	1	2
First Polar Body	-	fragmented	intact
Pervitelline Space	Large	-	normal
Cytoplasmic Granulation	present (spots, vacuoles, refractile body	-	absent

### Data analysis

Data of expression of LH-R gene, oocyte maturity, morphology, and fertilization rate were analyzed. The normality of the data was tested using Shapiro-Wilk test. Normally distributed data were then tested with the unpaired T-test, but if the data had an abnormal distribution, the Mann-Whitney test was performed to compare the differences in expression of LH-R gene between groups of poor responder and non-poor responder patients. Furthermore, the Spearman’s test was performed to determine the correlation between LH-R gene expression and oocyte maturity, morphology, and fertilization rate. A p-value of less than 0.05 was considered significant. Statistical analysis was performed using IBM SPSS (Statistical Package for Social Sciences) version 22.

## Results

Table 3 shows the characteristics of groups I and group II, poor and non-poor responders, respectively. We found that granulosa LH-R expression is higher in the poor responders (27.37 (0.00-28939.37) arbitrary unit) than in non-poor responders (0.00 (0.00-7196.12)), but this was not statistically significant between the groups (p=0.169).

**Table 3.** Patient characteristics of poor and non-poor responders to IVF in a group of patients in Jakarta.

Characteristics	Poor responder		Non-poor responder	
	n	Description	n	Description
Age (years; mean±SD)	10	37.80 ± 3.49	20	33.20 ± 3.61
AMH serum (ng/ml; mean±SD)	9	1.14 ± 0.76	15	4.64 ± 2.22
FSH total (IU/l; mean±SD)	10	3660 ± 854.99	20	2822.50 ± 783.88
E2 trigger (pg/ml; mean±SD)	10	1481.43 ± 721.12	20	2864.68 ± 1495.06
LH trigger (IU/l; mean 95%CI)	10	4.74 (1.04-23.28)	20	2.08 (1.00-6.60)
P4 trigger (ng/ml; mean 95%CI)	10	0.65 (0.20-0.80)	20	1.00 ± 0, 52
Total oocyte number (mean 95%CI)	10	4.50 (2.00-15.00)	20	14.95 ± 9.42
Oocyte maturity (mean 95%CI)	8	4.00 (2.00-13.00)	15	14.33 ± 7.07
Morphology (mean±SD)	8	4.08 ± 0.95	15	4.77 ± 0.65
Fertility rate (%; mean±SD)	10	55.00 ± 27.63	20	58.05 ± 24.56
Embryo transfer (mean 95%CI)	9	3.00 (1.00-3.00)	19	3.00 (1.00-4.00)
Clinical pregnancy (n (%))	Yes	2 (25.0)	Yes	10 (52.6)
	No	6 (75.0)	No	9 (47.4)

Table 4 shows a statistically significant negative correlation between oocyte maturity in the poor responders and granulosa LH-R expression (p=0.003; r=0.552). There is no correlation between granulosa LH- R expression and oocyte morphology. There is negative correlation between granulosa LH-R expression and fertility rate in the poor responders (p=0.215; r=0.430) and non-poor responders (p=0.142; r=-0.340), which was not statistically significant.

**Table 4.** Correlation between granulosa LH-R expression with maturity, oocyte morphology, and fertility rate of poor and non-poor responders to IVF treatment.

Characteristics	Granulosa LH-R expression	
	Poor responder	Non-poor responder
**Oocyte maturity**		
Correlation coefficient	0.267	-0.552
p value	0.523	0.033
n	8	5
**Morphology**		
Correlation coefficient	0.267	-0.164
p value	0.523	0.560
n	8	15
**Fertility rate** (%)		
Correlation coefficient	0.430	-0.340
p value	0.215	0.142
n	10	20

Raw data for all variables reported in the study Click here for additional data file. Copyright: © 2019 Wiweko B et al. 2019 Data associated with the article are available under the terms of the Creative Commons Zero "No rights reserved" data waiver (CC0 1.0 Public domain dedication).

## Discussion

From this study, granulosa LH-R expression in poor responders is higher than in non-poor responders, although it is not statistically significant. In a study by Thiruppathi *et al.* ^29^, which compared gonadotrophin receptor expression in poor responders and non-poor responders, the results also showed that granulosa LH-R expression is higher in poor responders. This may suggest that there is a disruption in LH-R processing and trafficking or it could be caused by accelerated release of LH in poor responders ^29^.

In this study, granulosa LH-R expression had a positive correlation with morphology, oocyte maturity and fertility rate in the poor responder group and a negative correlation in non-poor responder group. This showed that the poor responder group needs LH for oocyte growth and maturation, while in the non-poor responder group high granulosa LH-R expression would affect maturity and morphology of oocytes and fertility rate. Maman *et al.* ^18^ found that in non-poor responders, granulosa LH-R expression increased in the antral-phase follicle and the highest expression happened pre-ovulation. There was a correlation between LH-R expression and fertility output. In that study, low LH-R expression correlated with low oocyte maturity, but excessive LH-R expression correlated with a low fertility rate ^18^. From these studies, it seems that an optimal granulosa LH-R expression is needed to mature oocyte and yield a good fertility rate.

A literature study by Shoham ^19^ suggested that there is a therapeutic window with a threshold and ceiling in LH supplementation. If the LH level is below the needed ceiling, estradiol production will not be adequate, while if the LH level is higher than the threshold, there will be a negative impact on follicle growth. Shoham discovered that LH supplementation in patients with hypogonadotropic hypogonadism would generate more follicles and adequate estradiol levels to generate good endometrium growth. However, excessive LH supplementation in patients with hypogonadotropic hypogonadism or polycystic ovaries would cause negative effects, causing follicles to become atretic ^19^. The study is supported by Humaidan *et al.* ^29^, who found that LH level must be at an adequate level, not too high or too low, to generate good quality oocytes. Optimal LH levels measured in the 8th day of stimulation will decrease the required FSH doses, hence making the stimulation duration shorter and growth of good follicles faster ^29^. However, it must be remembered that this study was done in non-poor responder patients, using a GnRH agonist protocol, where patients’ gonadotropin is stimulated with GnRH agonist administration before stimulation. Therefore, if endogenous LH levels decrease too much because of that suppression, follicular growth will be disrupted, because the androgen production in theca cells, which will be converted into estradiol in granulosa cells, is decreasing. Because of that, non-poor responder patients who receive an agonist GnRH protocol should use an adequate dose that is not too high. Besides the dose, the administration mode also influences mid-follicular LH levels. In the administration of intranasal buserelin, the decrease of mid-follicular LH is not too low, and the pregnancy rate is better compared to subcutaneous administration ^29^.

In the present research, all subjects received the antagonist protocol, so there was no excessive endogenous LH suppression as seen in the agonist protocol. It seems that the stimulation protocol type may not influence granulosa LH-R expression. This is supported by a microarray analysis of gene expression study in rFSH and hMG stimulation, where LH-R expression showed no differences ^30^. Therefore, it may be inferred that the main contributors of granulosa LH-R expression are follicle size and follicle maturation stage ^18^. Granulosa LH-R is expressed in the early antral phase of follicle growth even when granulosa LH-R expression is still very low ^18, 31^. Then, by increasing follicle maturation under FSH influence, granulosa LH-R expression will also increase. Therefore, granulosa LH-R expression can be increased by exposing granulosa to adequate FSH before ^18^. However, how much FSH to get optimal LH-R expression must be investigated. The most common problem in poor responder patients, other than the inadequate number and bad quality of oocytes produced, is that the FSH dose needed for stimulation is too high. This has been shown in the present study ( Table 2). In this study, the total FSH dose used in the poor responder group was higher than that in the nonpoor responder group (3660 IU vs 2822 IU). In the Alviggi study, a higher FSH dose was also found in polymorphism LH patients (v-betaLH). According to Alviggi, homozygote and heterozygote v-betaLH patients have a poor ovarian response to gonadotropin and the need of total FSH dose is higher than in wild-type LH patients. The oocytes are also generated in a smaller number. In his hypotheses, Alviggi stated that it was caused by the difference in the bioactive effects between v-betaLH and wild-type LH ^25, 32^. v-betaLH has shorter t-half but has a more potent efficacy at receptor level compared to the wild-type ^25^. In the present study, analysis of v-beta LH was not done. Besides LH polymorphism, in the Alviggi study, there was also a patient who did not have v-betaLH but had an inadequate response to gonadotropin. The possible explanation of that phenomenon is due to the increment in FSH consumption without the presence of v-betaLH, which is caused by LH-R and FSHR polymorphisms ^32^. However, this still needs to be further investigated.

## Conclusions

This study showed that granulosa LH-R expression in poor responders is higher than non-poor responders. Statistical analysis showed a positive correlation between granulosa LH-R expression with oocyte quality and fertility rate in the poor responders, and a negative correlation between granulosa LH-R expression with oocyte quality and fertility rate in the nonpoor responders.

## Data availability

The data referenced by this article are under copyright with the following copyright statement: Copyright: © 2019 Wiweko B et al.

Data associated with the article are available under the terms of the Creative Commons Zero "No rights reserved" data waiver (CC0 1.0 Public domain dedication). 

*Underlying data* F1000Research: Dataset 1. Raw data for all variables reported in the study, https://doi.org/10.5256/f1000research.17036.d230859 ^33^
