# Supplementary material for: Correlation between luteinizing hormone receptor gene expression in human granulosa cells with oocyte quality in poor responder patients undergoing  in vitro fertilization: A cross-sectional study
Source: F1000Res. 2019 Jan 4;8:16. [Version 1] doi: 10.12688/f1000research.17036.1 (PMC6530604; doi:10.12688/f1000research.17036.1)
Supplement: Peer review history

# Peer review: Reviewer response for version 1

**DOI:** 10.5256/f1000research.18625.r47813

**Published:** 2019-05-17

**Authors:** Peng-Hui Wang

**Affiliations:** 1 Department of Obstetrics and Gynecology, Taipei Veterans General Hospital, Taipei, Taiwan; 2 Institute of Clinical Medicine, National Yang-Ming University, Taipei, Taiwan

The authors provided a cross section study to evaluate the predictor of oocyte quality 30 poor responders undergoing *in vitro* fertilization (IVF) and found the expression of luteinizing hormone receptor (LHR) was important to predict the oocyte quality of poor responders or non-poor responders, but the former is a positive correlation and the latter is a negative correlation. The current study showed oocyte quality could be predicted by oocyte-surrounding granulosa cells. Although the study is interesting, there are some issues worthy of further discussion.

1. For the definition of poor responders, it is very clear. The key factor of the criteria is a poor ovarian reservation (Chern *et al.*, 2018 ^1^, Li *et al.*, 2018 ^2^, Tsui *et al.*, 2017 ^3^, Lin *et al.*, 2017 ^4^, Lin *et al.*, 2017 ^5^, Lin *et al.*, 2017 ^6^, Lin *et al.*, 2015 ^7^, Tsui *et al.*, 2015 ^8^, Tsui *et al.*, 2014 ^9^). Even though there was an adequate protocol for ovary stimulation, the total number of the oocytes retrieved is limited. By contrast, high responders are often risky for the development of ovary hyperstimulation syndrome, and the total number of oocytes retrieved is too many. In clinical practice, polycystic ovary syndrome (PCO) might be one of the best examples. Although the ratio of LH/FSH is abandoned in the diagnostic criteria of PCO, we can predict the serum level of LH is higher in the high responders, which might be secondary to the relative “hyperandrogenism”. Although the authors used the non-poor responders as control, it is not clear that the similar finding could be applied in high responders.
2. The study material of granulosa cells might be interesting. Is there any difference of LHR expression between different populations of granulosa cells (oocyte surrounding granulosa cell (cumulus cells) or follicle-type granulosa cells)? (Lin *et al.*, 2017 ^4^, Tsui *et al.*, 2014 ^9^).
3. Since the significant difference of dosage of LH trigger between poor responders and non-poor responders was noted, it is rational to suppose the LHR expression will be different between two groups (Lin *et al.*, 2017 ^6^). The correlation between the ligand and receptor should be introduced in much more detail. Membrane-type receptor and nuclear-type receptor expression might be varied greatly or conflicted dramatically when the different dosage of ligands is provided. Down regulation and overexpression might be the secondary effect. In addition, if the oocyte quality is positively correlated with LHR expression in poor responders, the absolute qualification of the amount of LHR might be needed. It is relatively unusual that the same amount of the hormone profiles will result in the different clinical patterns. If this condition is real, how would the normal range of hormone profile in the clinical practice be obtained? The authors should consider it with much more concern.
4. Oocyte quality is often recognized by morphological classification. If more markers could be used in much more scientific or quantitative patterns, it is welcome. However, the easy-to-use rule and reproducibility might be important.

I have read this submission. I believe that I have an appropriate level of expertise to confirm that it is of an acceptable scientific standard.

## References

[1] ChernCUTsuiKHVitaleSGChenSNWangPHCianciATsaiHWWenZHLinLT: Dehydroepiandrosterone (DHEA) supplementation improves in vitro fertilization outcomes of poor ovarian responders, especially in women with low serum concentration of DHEA-S: a retrospective cohort study. *Reprod Biol Endocrinol*.2018;16(1) : 10.1186/s12958-018-0409-z 90 10.1186/s12958-018-0409-z 30223902PMC6142344

[2] LiCJChenSNLinLTChernCUWangPHWenZHTsuiKH: Dehydroepiandrosterone Ameliorates Abnormal Mitochondrial Dynamics and Mitophagy of Cumulus Cells in Poor Ovarian Responders. *J Clin Med*.2018;7(10) : 10.3390/jcm7100293 10.3390/jcm7100293 30241351PMC6210273

[3] TsuiKHWangPHLinLTLiCJ: DHEA protects mitochondria against dual modes of apoptosis and necroptosis in human granulosa HO23 cells. *Reproduction*.154(2) : 10.1530/REP-17-0016 101-110 10.1530/REP-17-0016 28624766

[4] LinLTWangPHWenZHLiCJChenSNTsaiEMChengJTTsuiKH: The Application of Dehydroepiandrosterone on Improving Mitochondrial Function and Reducing Apoptosis of Cumulus Cells in Poor Ovarian Responders. *Int J Med Sci*.2017;14(6) : 10.7150/ijms.18706 585-594 10.7150/ijms.18706 28638275PMC5479128

[5] LinLTWangPHChenSNLiCJWenZHChengJTTsuiKH: Protection of cumulus cells following dehydroepiandrosterone supplementation. *Gynecol Endocrinol*.2017;33(2) : 10.1080/09513590.2016.1214262 100-104 10.1080/09513590.2016.1214262 27684542

[6] LinLTsuiKWangP: The earlier the better: When should intrauterine insemination be done?. *Journal of the Chinese Medical Association*.2017;80(6) : 10.1016/j.jcma.2016.07.002 331-332 10.1016/j.jcma.2016.07.002 27595439

[7] LinLTTsuiKHWangPH: Clinical application of dehydroepiandrosterone in reproduction: A review of the evidence. *J Chin Med Assoc*.2015;78(8) : 10.1016/j.jcma.2014.12.008 446-53 10.1016/j.jcma.2014.12.008 25708822

[8] TsuiKHLinLTChangRHuangBSChengJTWangPH: Effects of dehydroepiandrosterone supplementation on women with poor ovarian response: A preliminary report and review. *Taiwan J Obstet Gynecol*.2015;54(2) : 10.1016/j.tjog.2014.07.007 131-6 10.1016/j.tjog.2014.07.007 25951716

[9] TsuiKHLinLTHorngHCChangRHuangBSChengJTWangPH: Gene expression of cumulus cells in women with poor ovarian response after dehydroepiandrosterone supplementation. *Taiwan J Obstet Gynecol*.2014;53(4) : 10.1016/j.tjog.2014.09.003 559-65 10.1016/j.tjog.2014.09.003 25510701

# Peer review: Reviewer response for version 1

**DOI:** 10.5256/f1000research.18625.r46684

**Published:** 2019-04-29

**Authors:** Yanping Kuang

**Affiliations:** 1 Reproductive Medicine Center, Shanghai Ninth Hospital, Shanghai Jiao Tong University, Shanghai, China

The aim of this study was to evaluate the role of LH and granulosa cell LH-R in poor responder patients who underwent COS. Then the authors analyzed the expression of LH-R in the granular cells from poor responder and non-poor responders and found the expression of LH-R mRNA had a positive correlation with oocyte quality in poor responder patients and a negative correlation in non-poor responders. The aim of this study was vague. The conclusion was not consistent with the purpose of this study and made with insufficient supporting data. The correlations between the LH-R expression and oocyte quality was established based on Spearman’s test analysis. However, the oocyte quality might be correlated with age, BMI, basal FSH level, basal LH level and other factors. To analyse the correlation between the expression of LH-R and oocyte quality, multivariate logistic regression should be performed to quantify the effect of all related factors on the oocyte quality.

I have read this submission. I believe that I have an appropriate level of expertise to confirm that it is of an acceptable scientific standard, however I have significant reservations, as outlined above.

# Peer review: Reviewer response for version 1

**DOI:** 10.5256/f1000research.18625.r46201

**Published:** 2019-04-01

**Authors:** Yuval Yung

**Affiliations:** 1 Reproduction Lab and IVF Unit, Department of Obstetrics and Gynecology, Sheba Medical Center, Tel Hashomer, Sackler Faculty of Medicine, Tel Aviv University, Tel Aviv, Israel

The manuscript by Wiweko *et al.* asks, does the LHR expression levels in mural granulosa cells correlate with embryo quality in poor/non-poor responder patients?

 Their results show a positive correlation between fertilization and embryo morphology in poor responder patients and a negative correlation between fertilization and embryo morphology in non-poor responder patients. These findings are novel and are well presented.

 One point of interest is missing in the discussion; LHR undergoes a sharp decrease after the LH surge (Nair *et al.,* 2002 ^1^ and Ophir *et al.,* 2014 ^2^) and the granulosa cells examined in this study were obtained around 34 h post LH surge when LHR levels should be under suppression. This may explain the lower LHR levels found in non-poor responders that responded better to the LH surge. Further discussion in this regard should be added.

 The manuscript after minor revision may be suitable for indexing.

I have read this submission. I believe that I have an appropriate level of expertise to confirm that it is of an acceptable scientific standard.

## References

[1] NairAKKashJCPeegelHMenonKM: Post-transcriptional regulation of luteinizing hormone receptor mRNA in the ovary by a novel mRNA-binding protein. *J Biol Chem*.2002;277(24) : 10.1074/jbc.M111653200 21468-73 10.1074/jbc.M111653200 11940568

[2] OphirLYungYMamanERubinsteinNYerushalmiGMHaasJBarzilayEHourvitzA: Establishment and validation of a model for non-luteinized human mural granulosa cell culture. *Mol Cell Endocrinol*.2014;384(1-2) : 10.1016/j.mce.2014.01.018 165-74 10.1016/j.mce.2014.01.018 24508664
